# The Relationship Between Basic Needs Satisfaction, Self-determined Motivation, and Burnout in Korean Esports Players

**DOI:** 10.1007/s10899-022-10132-8

**Published:** 2022-05-31

**Authors:** Hee Jung Hong, Guy Wilkinson, Claudio M. Rocha

**Affiliations:** Faculty of Health Sciences and Sport, University of Stirling, Stirling, FK9 4LA Scotland, UK

**Keywords:** Online games, Athlete burnout, Esports players, Self-determination theory, Esports players’ motivation

## Abstract

This article aims to understand the relationship between basic needs satisfaction, self-determined motivation, and burnout in esports players. To achieve this, we investigate three research hypotheses: (1) The three dimensions of basic needs satisfaction have a positive effect on the self-determined motivation of esports players, (2) The self-determined motivation of esports players has a negative effect on their burnout, and (3) All three dimensions of basic needs satisfaction affect esports player’s burnout, indirectly via self-determined motivation. Data were collected through an online questionnaire (n = 608) administered to Korean esports players who play online games as a leisure activity. Results indicate that player competence has a negative relationship with self-determined motivation, suggesting that Korean players do not associate increased levels of competence with their intrinsic motivation. The results show that intrinsic motivation is negatively associated with burnout, particularly exhaustion and reduced sense of accomplishment. It should be noted that Korean players’ high level of competence can result in reduced levels of self-determined motivation, which can lead them to burnout. This should be addressed by the industry, practitioners, and researchers considering the cultural context and the relationship between the factors, which will contribute to the sustainable growth and prosperity of the esports.

## Introduction

Researchers have paid increasing attention to esports, as it is a growing topic in both industry and academia. Pedraza-Ramirez et al. (2020) defined esports as “the casual or organised competitive activity of playing specific video games that provide professional and/or personal development to the player” (p. 6). They also pointed out that not all video games are considered as esports games, but all esports games are video games. The Pedraza-Ramirez et al. (2020) definition is applied in this study. While there has been a transition in the industry from recreational and unstructured practice/activities into a competitive high-performance structure where players professionally compete (Pedraza-Ramirez et al., 2020), esports has become an increasing popular, growing leisure activity around the world in the last decade (e.g., Boudreau & Consalvo, 2014; Scholz, 2019). Since young people are getting more engaged in esports and its industry has grown rapidly (Smith et al., 2019), it is important to better understand esports itself and investigate possible issues associated with it to better promote the sustainable growth of the industry and ensure young players’ health and wellbeing.

While esports is relatively a new topic in academia, there have been some studies highlighting either the positive or negative aspects of esports. It is considered important to have a balanced view on esports to investigate the emergent research topic. Firstly, literature has described some positive aspects of esports that are often overlooked, such as developing social networks and creating new relationship/ties (Frostling-Henningsson, 2009; Jansz & Tanis, 2007; Trepte et al., 2012). In a recent empirical study, esports players at different levels identified that they developed a strong social network with other players and valued social support from their gaming communities (Hong & Wilkinson, 2020). Carbonie et al. (2018) in their study of positive personal development through esports identified eight benefits and four value groups, and pointed out that such benefits and values are in relation to esports players’ wellbeing. Kardefelt-Winther (2017) also notes that many individuals tend to use gaming as a coping mechanism for some issues they faced such as underlying health-conditions. Nevertheless, esports might be still subject to concerns due to its close relation to general video gaming which is played online (online gaming). For instance, some researchers questioned if professional players that spend 10 h or more a day training and competing should be considered as addicted to gaming or work (Faust et al., 2013; Griffths, 2017). Although all video/online games are not esports games as mentioned earlier, the difference between esports gaming and video/online gaming is not always clear to the public, which leads them to think they are identical. Therefore, excessive hours of training and competing in esports games can be considered as a risk factor.

While different positive aspects of esports have been reported, there have been studies to demonstrate some risks of esports. Past research on esports have discussed its risks (Ng & Wiemer-Hastings, 2005; Peters & Malesky, 2008; Whang & Chu, 2007). Ng and Wiemer-Hastings (2005) points out that players who play massively multiplayer online role-playing games (MMORPGs) tended to spend many more hours on playing games than the ones who play games offline. Although the study highlights the negative aspect of esports, the fact that the players do not exhibit any problematic behaviours of addiction is against the usual criticism of esports – that online gaming is harmful because it can lead to gaming addiction (WHO, 2018). The evidence from Peters and Malesky’s (2008) study suggests that playing online games may not be the only reason that causes issues and problems in players’ lives. Rather, other aspects such as individual personality and social context may contribute to determine if a player is at high risk to develop psychological problems. However, researchers still raised an issue of excessive hours of training and competing as one of the risk factors of esports. Bányai et al. (2019) suggested the lifestyle of esports players that can be frenetic, irregular, and involves excessive hours of gaming which can negatively impact on their performance as well as their psychological wellbeing and lifestyle. DiFrancisco-Donoghue et al. (2019) demonstrated in their study of managing the health of esports players that collegiate esports players played esports games up to 10 h per day and are exposed to overuse injuries such as eye fatigue, back pain, wrist pain, and hand pain. Some authors also suggest that esports increases the risk of isolation, particularly for young people (Orleans & Laney, 2000) although other studies demonstrated that individuals could develop social skills and bond as mentioned above.

In the more recent studies, researchers investigated esports players’ sleep behaviour as a risk factor (Bonnar et al., 2019; Lee et al., 2020), which can influence their performance as well as both physical and mental health and wellbeing. Wei et al. (2012) report that esports players playing for longer hours tend to show depressive, social phobic, and internet addiction symptoms when compared to moderate players. In this regard, other researchers have raised concerns related to the psychological health of players due to excessive gaming and/or addiction such as anxiety (Lo et al., 2005) and depression (Parsons, 2005). Young (2009, p.358) mentions, “online gaming is an emotionally draining and time-consuming activity.” She insists that gaming addicts might suffer from several health problems such as back strain, eye strain, and repetitive stress injury that resulted in lack of rest and nutrition. Hong and Wilkinson (2020) reported that esports players at different levels found the psychological aspects of gaming very significant and they need more support for coping with psychological distress and ensuring their mental health. It was also reported that esports gaming can be very demanding psychologically as well as physically so that young players are exposed to experiencing burnout due to excessive hours of gaming (Lajkah, 2018). Such psychological issues have been discussed with the burnout syndrome in traditional sport, which is called ‘athlete burnout’ (Raedeke, 1997). Athlete burnout is another psychological problem which, like anxiety, can lead to mental health issues such as depression.

Athlete burnout has been defined as a syndrome determined by emotional and physical exhaustion, sport devaluation, and a diminished sense of accomplishment (Lonsdale et al., 2009). It is also considered as a maladaptive psychological distress in relation to sport participation caused by excessive practice in traditional sport (Smith, 1986). Athlete burnout suggests a negative evaluation of an individual’s self and sport experience (Lemyre et al., 2007). Self-determination theory (SDT—Deci & Ryan, 1985) has been applied to research to measure athlete burnout (Hodge et al., 2008; Lemyre et al., 2006). Self-determination theory (SDT—Deci & Ryan, 1985) explains how people attain their basic needs satisfaction of competence, autonomy, and relatedness. In the quest of attaining such needs, athletes may have relied on means that are not effective to improve performance (e.g., long hours of training with not enough rest), leading to problematic consequences such as the burnout syndrome (Hodge et al., 2008; Lemyre et al., 2006). The basic needs satisfaction theory is a sub-theory of SDT that people seek accomplishment in three areas—competence, autonomy, and relatedness—to feel satisfied with their lives (Deci & Ryan, 2000). In traditional sport, competence means a perception of an individual’s ability in their sport; autonomy refers to feelings of volition, choice, and self-directedness; and relatedness means perceptions of connectedness with other individuals such as teammates and coaches (Lonsdale et al., 2009). Individuals can experience satisfaction and wellbeing when such basic needs are met (Ryan & Frederick, 1997). Contrarily, they may develop psychological problems, such as burnout, if the basic needs are not satisfied (Perreault et al., 2007). In self-determined motivation (Deci & Ryan, 1985), there are three different types of motivation: amotivation (a lack of motivation), extrinsic motivation (driven to achieve separable outcomes), and intrinsic motivation (driven by one’s own interest or enjoyment) (Lonsdale et al., 2009). Findings from the research in high-performance sport indicates that needs satisfaction is positively associated with fostering self-determined motivation (e.g., Hollembeak & Amorose, 2005). This will lead to some positive psychological consequences, such as adaptive coping (Amiot, et al., 2004) and flow experiences (Kowal & Fortier, 1999). Thus, athletes whose needs are not met demonstrate greater amotivation, which leads to maladaptive consequences such as dropping out of sport (Sarrazin et al., 2002).

Originally developed to explain physiological and psychological problems in athletes, burnout has recently been applied to professional esports players (Pérez-Rubio et al., 2017). Emotional and physical exhaustion are in relation to the high demands of training and competing (Lemyre et al., 2007), which is closely associated with the esports players’ experience (e.g., Zhuang, et al., 2020). The literature has not yet applied such a framework to the context of burnout in esports as a leisure activity. It may be expected that similar patterns develop in athletes, in their quest to attain basic psychological needs. As Young (2009) describes that online gaming can cause emotional exhaustion, the measure of player burnout in the present study is well-suited in relation to esports players’ psychological health and well-being. We use the term of ‘*player burnout*’ derived from athlete burnout, as we appreciate some commonalities between traditional sport and esports (Hallmann & Giel, 2018) but allow for a distinction for the burnout of esports players compared to athletes in traditional sport.

The aim of this study is, therefore, to explain the relationship between basic needs satisfaction, self-determined motivation, and burnout in Korean esports players. To achieve this aim, we propose three hypotheses. Hypothesis 1 is divided in three sub-hypotheses because it refers to the relationship between the three dimensions of basic needs satisfaction and self-determined motivation. Hypothesis 2 is divided in two sub-hypotheses, because it refers to the relationship between self-determined motivation and two dimensions of burnout.

- **H1a** Autonomy has a positive effect on self-determined motivation of esports players.
- **H1b** Competence has a positive effect on self-determined motivation of esports players.
- **H1c** Relatedness has a positive effect on self-determined motivation of esports players.
- **H2b** Self-determined motivation of esports players has a negative effect on reduced accomplishment of players.
- **H3** All three dimensions of basic needs affect esports player’s burnout, indirectly via self-determined motivation.

## Method

### Participants

The participants were young adults who play esports in South Korea. The esports industry has been growing rapidly in the East Asian region (e.g., South Korea, China, Japan, etc.) (Lee & Chung, 2021). Therefore, it is important to appreciate how esports may have led to a new type of social interaction and engagement in the region (Fung & Ho, 2015). Such an understanding of esports within the South Korean context will also lead us to capture the experience of players practicing esports as a popular leisure activity (Hjorth & Chan, 2009). Furthermore, Korean and Asian American players represent the largest number of players around the world (O’Neill, 2014). Therefore, Koreans who play esports as a leisure activity represent the target population of this study.

There is an approximately equal number of participants identifying as female (49.3%) and male (43.3%) in the sample. The respondents (n = 608) were mostly single (83.7%), with a high school level of education (63.7%), and aged 23.1 (*SD* = 5.8) years old on average. Most participants were undergraduate students (73.5%) and played 6.81 (*SD* = 13.2) hours of esports per week. Although extrapolations to the whole population are not allowed, this subset of the population is important to the esports industry because this is the demographic who primarily participate in esports. The most popular games played by participants included player versus player shooter PlayerUnknown’s Battlegrounds (16.3%) and multiplayer online battle arena League of Legends (13.5%), both prominent esports titles.

### Measurements

Basic Needs Satisfaction was defined based on Deci et al. (2001) scale. We follow them and define basic needs satisfaction by three first order latent variables: *autonomy* (7 items), *relatedness* (8 items) and *competence* (6 items). After running a first confirmatory factory analysis (CFA), the model was refined, and some items were dropped. This changed the *competence* variable to consist of 4 items for the data analysis. The stem for items in all three basic needs scales reads, “Please indicate how true each of the following statements has been for you, given your experiences on playing esports last year. When I play esports” (see Table 1 for all item wordings). The items were measured on a 7-point Likert scale, ranging from 1 (*not true at all*) to 7 (*very true*).

**Table 1:** Factors, item wordings, factor loadings (λ), average variance extracted (AVE), Cronbach’s alphas (α) and descriptive statistics (M and SD)

Scales and item wordings	λ	AVE	α	*M*	*SD*
*Basic needs satisfaction (1-not true at all—7-very true)—when i play esports*					
Autonomy		0.592	0.897	3.88	1.36
I feel like I can make a lot of inputs to deciding how to play	0.718				
I feel relaxed	0.778				
I am free to express my ideas and opinions on playing games	0.834				
When I am playing games, I do what I think is correct to do	0.774				
My feelings are taken into consideration	0.700				
I feel I can pretty much be myself	0.749				
There is opportunity for me to decide for myself how to go about playing games	0.822				
Relatedness		0.706	0.937	4.06	1.44
I really like the people I play with	0.822				
I get along with people who I play with	0.866				
I pretty much like interacting with others when I play	0.880				
I consider the people I play with to be my friends	0.804				
People who I play with care about me	0.837				
There are many people who I play with that I am close to	0.804				
The people who I play with seem to like me much	0.867				
People who I play with are pretty friendly towards me	0.837				
Competence		0.806	0.927	3.57	1.55
I feel very competent	0.906				
People who I play with tell me I am good at what I do	0.874				
When I am playing games, I get much of a chance to show how capable I am	0.930				
When I am playing, I often feel very capable	0.879				
*BURNOUT (1- almost never—5-almost always)*					
Exhaustion		0.732	0.877	2.07	0.97
I feel overly tired from playing games	0.784				
I feel "wiped out" from playing games	0.861				
I feel physically worn out from playing games	0.875				
I am exhausted by the mental and physical demands of playing games	0.899				
Reduced accomplishment		0.663	0.683	2.53	0.97
I am not accomplishing many worthwhile things in playing games	0.789				
I am not achieving much in playing games	0.820				
I am not performing up to my ability in playing games	0.834				
*Self-determined motivation (1-not true at all—7- very true)—i participate in esport*					
Intrinsic motivation		0.853	0.958	4.50	1.75
Because I enjoy it	0.931				
Because I like it	0.943				
Because it's fun	0.947				
Because I find it pleasurable	0.872				
Integrated Regulation		0.741	0.920	2.79	1.49
Because it's a part of who I am	0.792				
Because it's an opportunity to just be who I am	0.850				
Because what I do in sport is an expression of who I am	0.895				
Because it allows me to live in a way that is true to my value	0.901				
Identified regulation		0.832	0.952	2.68	1.51
Because the benefits of sport are important to me	0.909				
Because it teaches me self-discipline	0.920				
Because I value the benefits of playing games	0.914				
Because it is a good way to learn things which could be useful to me in my life	0.906				
Introjected Regulation		0.807	0.945	2.25	1.44
Because I would feel ashamed if I quit	0.876				
Because I would feel like a failure if I quit	0.885				
Because I feel obligated to continue	0.888				
Because I would feel guilty if I quit	0.943				
External regulation		0.865	0.963	2.13	1.47
Because if I don’t other people will not be pleased with me	0.950				
Because I feel pressure from other people to play	0.947				
Because people push me to play	0.938				
To satisfy people who want me to play	0.884				

Burnout was defined based on Raedeke and Smith (2001). We decide to use two first order latent variables to represent burnout: *exhaustion* (5 items) and *reduced accomplishment* (5 items). Again, an initial CFA reduced the number of items in the model. The *exhaustion* variable was reduced to contain 4 items for the data analysis and the *reduced accomplishment* variable contained 3 items. The stem for items in the burnout scale reads, “Please indicate how often you have experienced the following feelings, based on your experiences on playing esports last year” (see Table 1 for all item wordings). The items were measured on a 5-point Likert scale, ranging from 1 (*almost never*) to 5 (*almost always*).

Self-determined motivation was measured using Lonsdale et al. (2008) Behavioural Regulation in Sport Questionnaire (BRSQ). BRSQ (Lonsdale et al., 2008) has been widely used in studies related to SDT in sport context (e.g., Giles et al., 2020; O’Neil & Hodge, 2020), which was considered suitable for the present study. They proposed a self-determined motivation index, based on five dimensions of motivation: intrinsic motivation, integrated regulation, identified regulation, introjected regulation, and external regulation. We calculated the self-determined motivation index based on Lonsdale et al. (2009) procedures. The self-determined motivation index was calculated using weighted scores from the subscales intended to represent different types of behaviour regulations. This BRSQ item scores are weighted as follows: −2*external regulation; −2*introjected regulation; 1*identified regulation; 1*integrated regulation; 2*intrinsic motivation. In total, we have 21 items. The stem for all items in the motivation scale reads, “Please indicate how true each of the following statements has been for you, given your experiences on playing esports last year. I play esports because” (see Table 1 for all item wordings). The items were measured on a 7-point Likert scale, ranging from 1 (*not true at all*) to 7 (*very true*).

The last section of the questionnaire contains demographic variables (age, gender, marital status, and education), time playing esports (in hours; participants answered the question: How many hours do you spend on playing esports per week?), and physical activity habits (in days; participants answered the question: During the last 7 days, on how many days did you do moderate/vigorous physical activities?).

### Procedures

The online questionnaire was initially developed in English and then translated to Korean by the first author who is bilingual. The translated questionnaire was reviewed by two South Korean PhD students who were studying their PhDs in the UK at the time of developing the questionnaire to ensure the reliability of the questionnaire. The online questionnaire was tested with five Korean students before it was launched to identify any flaws and miscommunication. No major differences were found between the first and the final English versions of the questionnaire, indicating that the integrity of all items was maintained during the translation process.

The questionnaire was distributed through the first author’s network in South Korea following institutional ethical approval from the General University Ethics Panel (GUEP). The study complies with all regulations and confirmation that informed consent was obtained. The first author contacted three people from her personal network who have a strong network with undergraduate students, sport clubs, social communities and agreed to share the online survey with people who play esports. Snowball sampling was applied afterwards. Those initial contacts also reached out other groups of people who met the criteria via their own networks. The participants were informed about the study including the purpose of study. They were asked to confirm that they are over 18-year-old and agreed to participate in this study voluntarily.

### Data Analysis

A covariance-based structural equation modelling (SEM) analysis was conducted, following the two step-approach (Anderson & Gerbing, 1988), using Mplus 7.11. In the first step, the measurement model was tested using confirmatory factor analysis (CFA) technique. In the second step, the structural model, using SEM. Three indices were used to assess the model fit: root mean square error of approximation (RMSEA—values equal to or less than 0.05 and 0.08 indicate respectively a close and a reasonable fit; Hu & Bentler, 1998), comparative fit index and Tucker-Lewis index (CFI and TLI—values higher than 0.90 indicate close fit; Hair et al., 2009; Hu & Bentler, 1998). Using the measurement model, the constructs’ reliability (internal consistency) was indicated by the measures of Cronbach’s alpha (α). Values above 0.60, preferably above 0.70, are considered adequate for scales used in social studies (Bagozzi & Yi, 1988; Nunnally & Bernstein, 1994). Average variance extracted (AVE) was used as an indicator of convergent validity (Fornell & Larcker, 1981). A value of at least 0.50 indicates that the variance due to measurement error was smaller than the variance explained by the construct’ indicators (items). The Wald chi-square test for parameter equalities was applied to test correlations between pairs of constructs, which should be significantly different from one to indicate discriminant validity (Bagozzi et al., 1991; Guo et al., 2008).

Following Lonsdale et al. (2009), to test the measurement model, we ran two CFA – one for scales in basic needs satisfaction (autonomy, competence, and relatedness) and in burnout (exhaustion and reduced accomplishment) and another for the self-determined motivation scale. In the structural model, we tested whether self-determined motivation mediates the relationship between basic needs satisfaction and burnout. To test the structural relationships, we use three control variables: gender (0 = male; 1 = female), time playing esports (in hours), and physical activity habits per week (in days). We estimate indirect effects using the product-of-coefficients strategy (Sobel, 1982) with the multivariate delta method (Preacher & Hayes, 2008), available in Mplus 7.11.

## Results

Descriptive statistics show that, on average, Korean esports players do not have high degrees of autonomy (*M* = 3.88; *SD* = 1.36), relatedness (*M* = 4.06; *SD* = 1.44) and competence (*M* = 3.57; *SD* = 1.55) – the dimensions of basic needs satisfaction – when they play. On average, they report autonomy and competence below the mid-point 4, while they report relatedness slightly above the mid-point 4, in a 7-point Likert scale. In the burnout dimensions, players report low levels of exhaustion (*M* = 2.07; *SD* = 0.97) and reduced accomplishment (*M* = 2.53; *SD* = 0.97). In both scales, they are below the mid-point 3, in a 5-point Likert scale.

Results of the CFA with dimensions of basic needs satisfaction and burnout show that the measurement model fit the data acceptably well (CFI = 0.968; TLI = 0.964; RMSEA [90% CI] = 0.077 [0.073; 0.081]). Results of the CFA with self-determined motivation dimensions show that the measurement model fit the data acceptably well (CFI = 0.955; TLI = 0.947; RMSEA [90% CI] = 0.077 [0.072; 0.082]). Items’ wordings, factor loadings, AVE and Cronbach’s alphas are presented in Table 1. The Cronbach’s alphas are all above 0.70, showing good internal consistency for all measures in the scales (Nunnaly & Bernstein, 1994). Higher values of alpha may indicate some item redundancy. Deleting some items might drop the values of alphas. However, we decided to keep all the items as they come from original scales in the literature (Deci et al., 2001; Lonsdale et al., 2008; Raedeke & Smith, 2001).

The AVE of all scales are above 0.50, indicating large factor loadings and implying convergent validity. The results of the Wald chi-square test for parameter equalities indicate that none of correlations between pairs of constructs was equal to one, supporting discriminant validity (Guo et al., 2008).

**Fig. 1:**
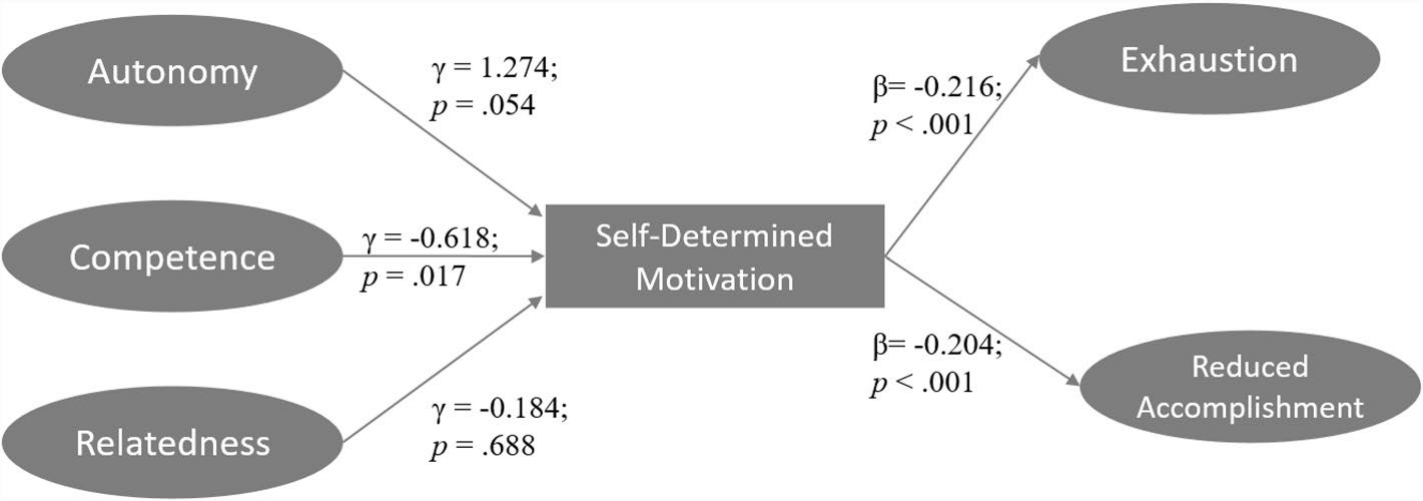
Structural relationships among dimensions of basic needs satisfaction (autonomy, competence and relatedness), self-determined motivation and dimensions of burnout (exhaustion and reduced accomplishment)

Results of the SEM analysis show that the structural model fit the data acceptably well (CFI = 0.914; TLI = 0.904; RMSEA [90% CI] = 0.065 [0.061; 0.070]). In the structural model (Fig. 1), the path coefficients from autonomy (γ = 1.274; *p* = 0.054) and relatedness (γ = − 0.184; *p* = 0.688) to self-determined motivation were non-significant; but the path coefficient from competence to self-determined motivation was negative and significant (γ = − 0.618; *p* = 0.017). The path coefficients from self-determined motivation to both exhaustion (β = − 0.216; *p* < 0.001) and reduced accomplishment (β = − 0.204; *p* < 0.001) were negative and significant. The indirect effect estimates from competence via self-determined motivation to exhaustion (IND = 0.133; *p* = 0.036) and to reduced accomplishment (IND = 0.126; *p* = 0.043) were both significant. However, all other four indirect effects were not significant: from autonomy to exhaustion (IND = − 0.275; *p* = 0.078) and to reduced accomplishment (IND = − 0.259; *p* = 0.085) and from relatedness to exhaustion (IND = 0.040; *p* = 0.689) and to reduced accomplishment (IND = 0.037; *p* = 0.690).

## Discussion

The popularity of esports originated from Asia where South Korea may be the most involved country (Wingfield, 2014). Adamus (2012) points out that South Korea is the centre of esports in Asia and highlights that playing esports has become a professional career in Korea. The growth of esports’ popularity has sparked off from Asia, and South Korea was the leading country in the phenomenon (Jenny et al., 2017). Although the market in North America and China has hugely grown over the last decade, South Korea is still one of the leading countries in the esports industry across the world (Episkopos, 2020). In particular, the International Esports Federation (IeSF) that has 98 national members across five continents is based in South Korea. Seo, (2013) demonstrates that the Korean broadband infrastructure has increasingly grown as Korean policymakers had relaxed controls in the mid-nineties. This enhanced infrastructure has been mainly used via digital television and online gaming (Wagner, 2006), which led to the increasing growth of esports.

The esports industry is a global phenomenon that increasingly attracts individuals as both players and spectators (Hallmann & Giel, 2018). While some researchers found benefits such as creating offline social capital (Trepete et al., 2012), increased self-esteem and personal satisfaction (Demetrovics et al., 2011), and a sense of accomplishment and social recognition (Seo, 2016), other researchers identify some negative aspects of playing esports such as being exposed to physiological stresses (Rudolf et al., 2016), increasing the risk for isolation (Orleans & Laney, 2000), and negative influence on their psychological wellbeing and daily life (Banyai et al., 2019). The present study provides empirical evidence to understand the relation between esports players’ basic needs, self-determined motivation and burnout by testing three hypotheses. The path coefficients from *autonomy* and *relatedness* to self-determined motivation (intrinsic motivation) were insignificant, providing a lack of support for H1a and H1c. The findings in this study do not support H1b as the path coefficient from *competence* to self-determined motivation (intrinsic motivation) was significant and negative. This can be interpreted that only a players’ competence relates to their intrinsic motivation to play esports and there is a negative relationship between them. *Competence* is the need of being effective when an individual interacts with the environment (Deci, 1975); within the esports context, it can refer to one’s perception on the ability to effectively play games. The reasons why the South Korean players’ competence was significantly and negatively associated with intrinsic motivation can be suggested based on their culture. Competence of South Koreans has been mainly measured in relation to education. South Korea is a competitive society where academic performance is highly emphasised because the society believes that it will make a great contribution to a successful life by entering a highly ranked university, securing a better job, and developing better networks (Lee, 2013). The Straits Times (2017) reported that 54 percent of Korean adults who participated in a survey indicated that they considered leaving Korea to live abroad; one of the reasons is the ‘*fierce competition*’. In the article, it also presented an interview from one 30-year-old Korean student saying, “I think that the competition in Korean society is not something I can get over through efforts (para. 16)”. In turn, developing one’s competence can be considered as essential to be better than others to survive the pressure from the society. Therefore, it can be said that it may be hard for Korean players to associate their increased competence with intrinsic motivation. This leads us to understand why H1a and H1c are insignificant. We speculate that since the social pressure is so strong, it may dominate over the effects on intrinsic motivation that players’ autonomy and competence provide. Ryan et al. (2006) investigate motivation for playing computer games and effect of playing games on players’ wellbeing. The findings show that the three items of basic psychological needs respectively indicate players’ enjoyment and future participation in gaming. The basic needs theory (Deci & Ryan, 2000) highlights that an individual’s experience that meets three basic needs contributes to enhancing one’s wellbeing; and intrinsic motivation is associated with gaming participation that most players are not driven by external reward or approval (Ryan et al., 2006). This study suggests that in the case of South Korea, only competence indicates a player’s motivation to play games and there is a negative relationship between them, reflecting cultural differences from previous studies.

There is support for both H2a and H2b as the path coefficients from self-determined motivation to both exhaustion and reduced accomplishment were significant and negative. Within sport context, some researchers found that intrinsic motivation was negatively associated with athlete burnout (Raedeke & Smith, 2001), which is confirmed by the findings in this study. Researchers demonstrate that self-determined motivation—particularly amotivation and intrinsic motivation—has a strong and consistent relation to athlete burnout (e.g., Cresswell & Eklund, 2005; Lonsdale et al., 2009). The findings in this study provide evidence to support this that intrinsic motivation is negatively associated with burnout, particularly exhaustion and reduced accomplishment. This suggests that Korean players’ enjoyment of playing games is related to lower levels of player burnout, which should be highlighted as a key aspect of esports being widely played as a leisure activity. Korean PC Bangs (rooms), which refers to a cybercafé where young people play online games with low cost, play an important role in “shaping and forming the sociocultural context” (Huhh, 2008, p. 27). It represents an important social space where they can establish online/offline communities (Chee, 2006) and space for entertainment, which is a crucial part of a Korean’s socialising environment (Stewart & Choi, 2003). Therefore, the aspects of social interaction and entertainment from playing esports should be more emphasised for players’ health and wellbeing than ‘competitiveness’.

H3 is supported in part by the finding that the indirect effect estimates from competence via self-determined motivation to exhaustion and to reduced accomplishment were both significant. The value of these two indirect effects is positive because this is the product of two negative values; one from competence to self-determined motivation, and the other from self-determined motivation to exhaustion (the same is true for reduced sense of accomplishment). High levels of competence lead to reduced levels of self-determined motivation, which in turn leads to higher levels of exhaustion and reduced sense of accomplishment. Therefore, indirectly, through self-determined motivation, higher feelings of competence lead to higher levels of exhaustion and reduced sense of accomplishment. Within sport context, researchers found that autonomy and competence were significant predictors of athlete burnout (Hodge et al., 2008), which was confirmed by Lonsdale et al. (2009). The findings in this study also support their findings in part as only competence was negatively associated with burnout. However, it is worth noting that the findings in both sport and esports are shared. Lonsdale et al. (2009) found that the relationships between the basic needs and exhaustion and devaluation were highly mediated by self-determined motivation, whilst their relationships with reduced sense of accomplishment was only partially mediated, which is partly supported by the findings in current study. Therefore, in relation to burnout, players’ intrinsic motivation can reduce a chance for players to experience burnout, but our study shows that contrary to previous research, in the case of South Korea, external cultural factors can produce a negative indirect effect between competence and burnout. This unexpected result indicates that it is important to understand esports within specific cultural contexts, which is still limited in esports studies. This finding also indicates that the industry should address possible issues like burnout, which may cause maladaptive consequences such as dropping out of the practice (Sarrazin et al., 2002), to sustain its growth.

## Conclusion

The current study presents empirical evidence to understand the relationship between esports players’ basic needs, self-determined motivation, and burnout. The findings show that player competence has a negative relationship with self-determined motivation suggesting that Korean players do not associate increased levels of competence with their intrinsic motivation, which challenges the previous findings in the literature. We argue that Korean players may be more externally motivated than internally motivated to play games. We also suggest that competitive aspects within Korean culture as the likely cause for this relationship, as motivation may be sought externally through direct competition with their peers. Regarding the competitive culture, this may be one of key reasons that South Korea has been recognised as a hub of esports and could be successful to cultivate talented players who compete at high level. Therefore, the current study gives an insight into future studies related to burnout and self-determination theory in that more studies should be done in different cultural contexts to better understand esports players’ experience. This should, in turn, inform the esports industry and relevant practitioners to develop customised and evidence-based support services to protect them from burnout. It should be noted that South Korean players’ high level of competence can result in reduced levels of self-determined motivation, which can lead them to burnout. This, again, should be addressed by the industry, practitioners, and researchers considering the culture context and the relationship between the factors, which will contribute to the sustainable growth and prosperity of the esports.

There are some limitations presented in this study, which should be considered for future studies. We adopted a cross-sectional design, so no causal inferences can be concluded from our findings. The use of a convenience sample means that the results of this study cannot be extrapolated to the whole population of esports players in South Korea or/and esports players in other countries. However, this evidence does provide some insight into a research area which has not previously been explored. While it is hard to demonstrate that the results can be applied to other players in different cultural contexts, esports players’ burnout should be prevented to ensure their overall wellbeing. Therefore, it is important for esports players themselves and other stakeholders to be aware of this issue and to develop corresponding action plans. Future research should target esports players in different cultures and at different levels (e.g., professional and semi-professional) to better understand their influence on the relationship between competence, motivation, and burnout. Only two indicators (emotional and physical exhaustion and a diminished sense of accomplishment) were used to measure player burnout. Future studies may consider using all three including sport devaluation to have a broader understanding of burnout that esports players experience.
